# Supplementary figures and images for: The post-septic peripheral myeloid compartment reveals unexpected diversity in myeloid-derived suppressor cells
Source: Front Immunol. 2024 Apr 24;15:1355405. doi: 10.3389/fimmu.2024.1355405 (PMC11076668; doi:10.3389/fimmu.2024.1355405)

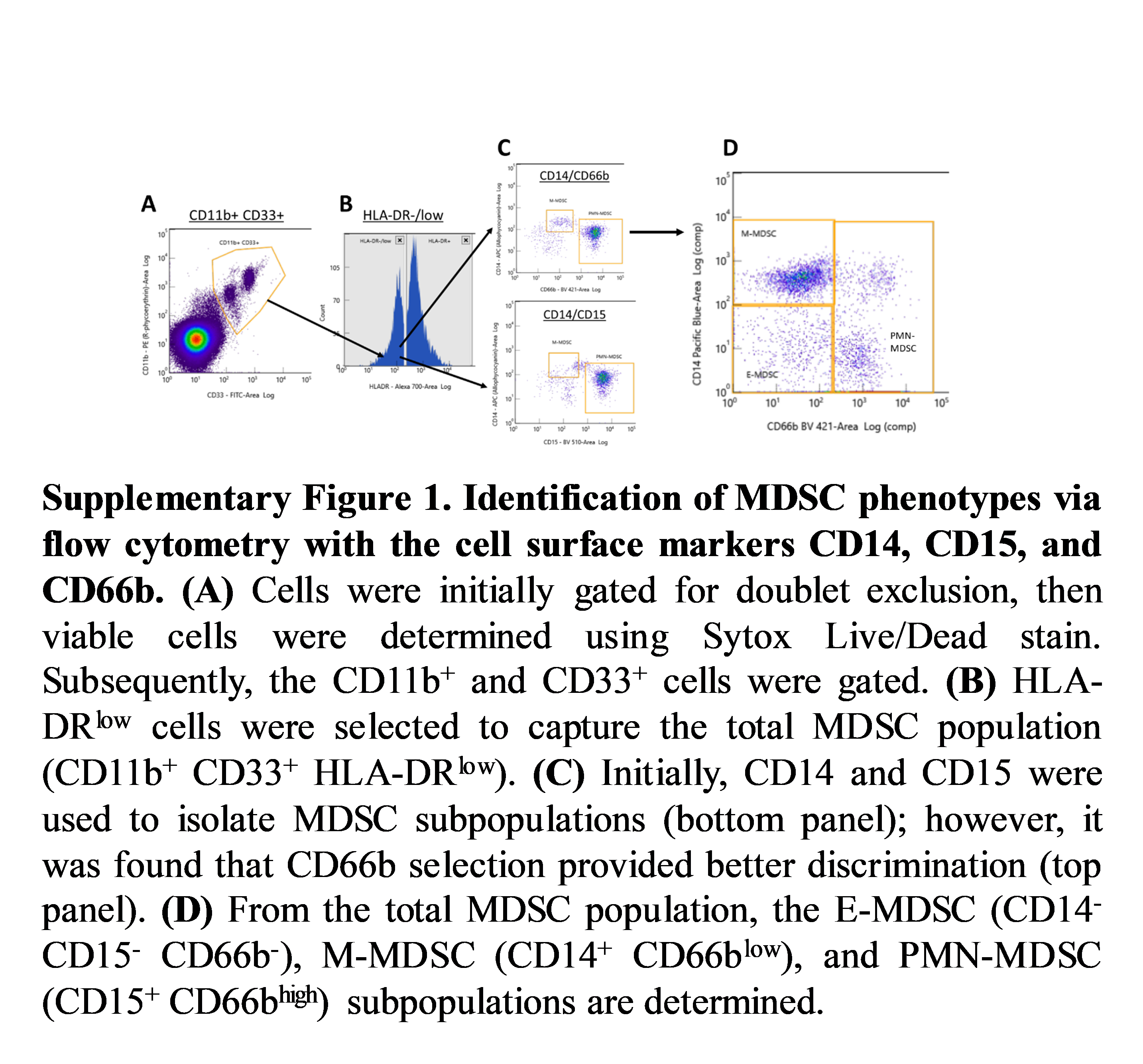

Supplement: Supplementary file 1 [file Image_1.tif]

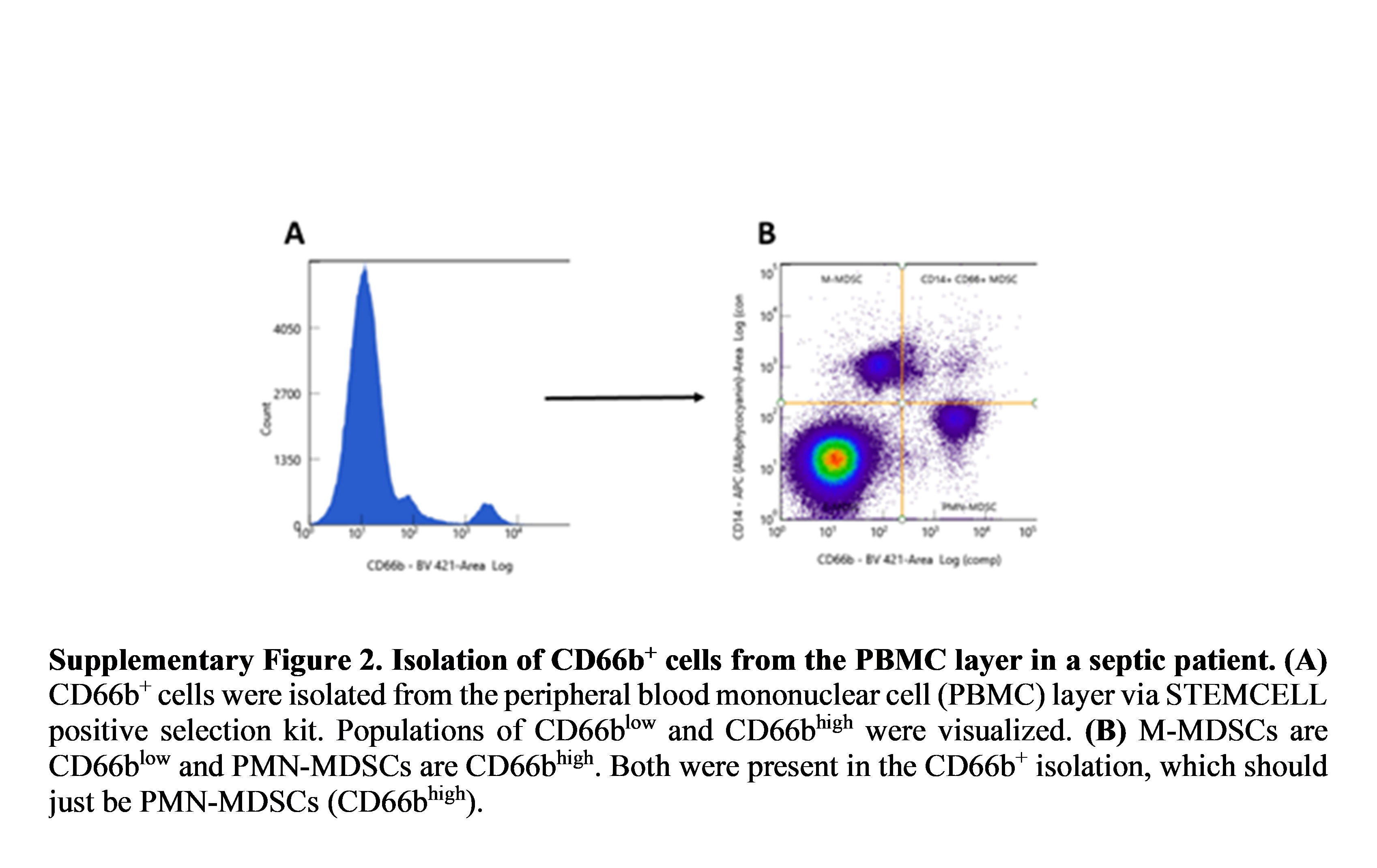

Supplement: Supplementary file 2 [file Image_2.tif]

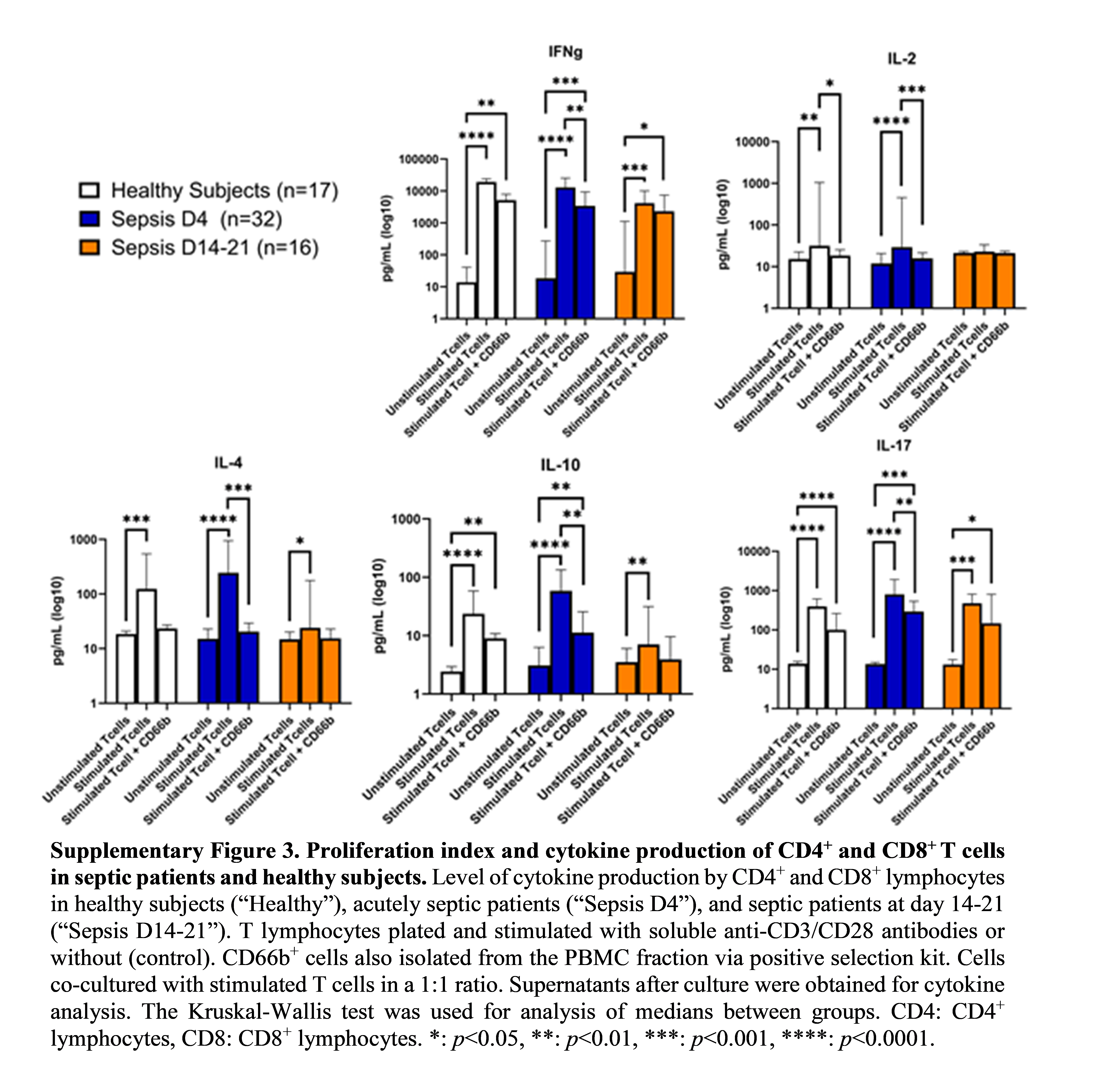

Supplement: Supplementary file 3 [file Image_3.tif]

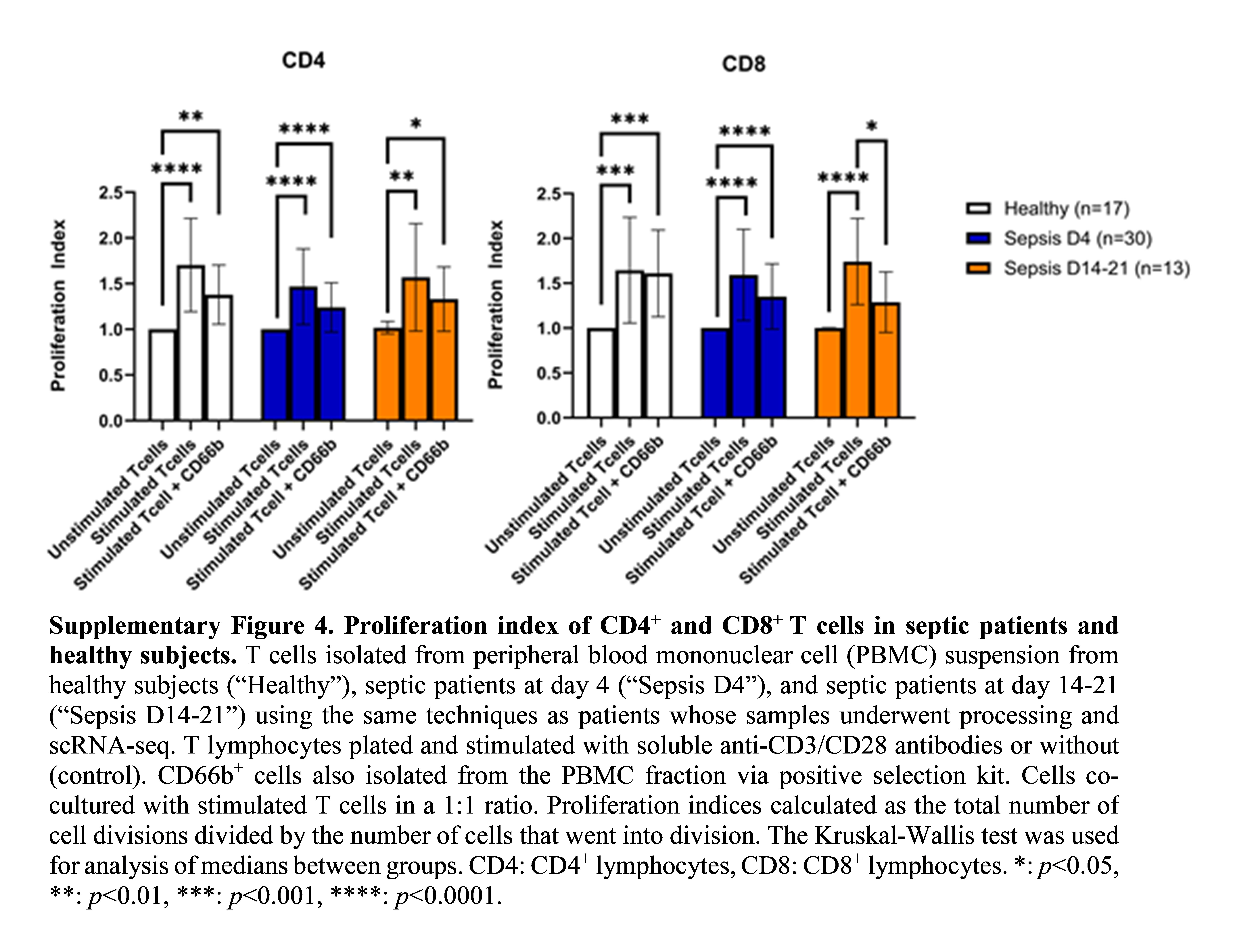

Supplement: Supplementary file 4 [file Image_4.tif]

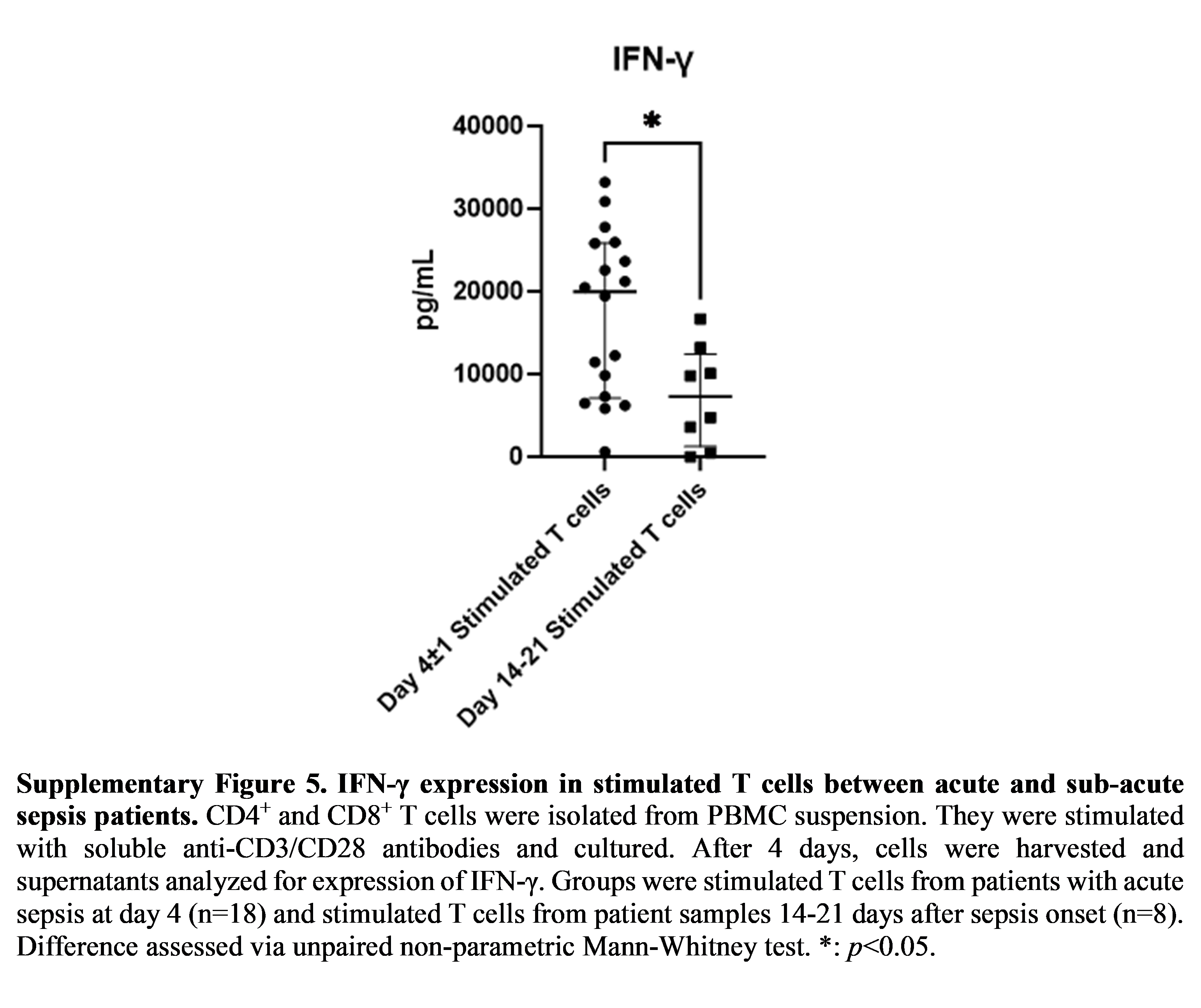

Supplement: Supplementary file 5 [file Image_5.tif]
